# Definitions of Solitude in Everyday Life

**DOI:** 10.1177/01461672221115941

**Published:** 2022-09-03

**Authors:** Netta Weinstein, Heather Hansen, Thuy-vy Nguyen

**Affiliations:** 1 University of Reading, UK; 2 Durham University, UK

**Keywords:** solitude, alone, quiet, balance, leisure

## Abstract

What does it mean to be in solitude? Researchers building this nascent field are learning much about the potential affordances of solitude, but lack an agreed-upon definition or set of definitions. Arriving at that meaning is crucial to forming a solid foundation for studies that use both naturalistic and laboratory designs to explore outcomes of solitude. This study identified themes from semi-structured interviews with adults aged 19 to 80 from diverse backgrounds. We concluded that solitude is a state in which the dominant relationship is with the self. If not physically alone, people in solitude are mentally distanced from others and away from active technology-mediated interactions. Complete solitude involves both physical separation and inner focus, but solitude is best defined through a taxonomy that recognizes physical separation and internal focus as independent, sufficient characteristics. An internal focus benefits from (but is not defined by) balancing solitude with social time, quiet, and choice.

## States of Positive Solitude in Everyday Life

Researchers and laypeople alike are coming to understand that along with their social pursuits, solitude—time alone—is a common and consequential part of daily life. In recent years, there has been evermore research exploring the topic from social, psychological, developmental, and personality perspectives (R. C. Coplan et al., 2021). This body of work is based on rich traditions within psychology, stemming from a psychodynamic theory that solitude presents a challenge to engage the self (Winnicott, 1958) and earlier from spiritual and hermetic traditions outside of psychology (Storr, 1988). There is vast opportunity for the growing body of research to pursue a deeper understanding of the benefits and costs of solitude; who benefits from or suffers in solitude and when; and how to intervene to promote positive solitude. To achieve this, the nascent literature on solitude must grapple with two main challenges: First, there is a continued need for unifying, agreed-upon definitions of solitude to study it systematically as a meaningful phenomenon and to communicate those findings. This definition is made evermore complex because the presence of devices that offer opportunities for virtual social interactions means we cannot simply reduce solitude to physical separation (Campbell & Ross, 2022). Second, we must recognize a major assumption about the nature of solitude—that it plays a substantive role in daily lives as a state that is in *itself* meaningful, regardless of whether or not the subjective experience of that state is positive or negative (R. J. Coplan et al., 2019). To build an understanding of solitude based on recognition of these challenges, the current project was designed to examine the nature of daily solitude by exploring its definition(s) in the context of daily life. We used a qualitative method and thematically analyzed in-depth interviews with individuals to form a conceptual model not bound by assumptions made in the existing literature—that is, we took an inductive, “bottom-up” approach (Patton, 2005) to the data.

### Definitions of Solitude

Solitude can be conceptualized as “being alone,” but this understanding does not constitute an operational definition, and the literature is mixed regarding how to approach a nuanced and specific definition of solitude. Prominent views include those of Larson (1990), who argued that solitude refers strictly to a physical and virtual separation from others that eliminates immediate social and interpersonal cues. Offering a different perspective, Long et al. (2003) argued that physical separation is not necessary provided there is no social interaction. Consider as an example “dinner for one” at a restaurant. Others are present, yet one is arguably in solitude—in their own space and not responding to others’ continual social cues and expectations. Building on this distinction, Nguyen et al. (2021) delineate “private” solitude that is physically removed from others from “public” solitude where others are present but not actively interacting. The authors also argued that we do not yet know whether others’ presence influences individuals’ thoughts and behaviors while in public solitude, or the extent to which that state is qualitatively different from private solitude, where one is physically separate from others. The importance of the distinction rests on a number of factors, including whether solitude is best understood as a physical state where physical aloneness is essential (and therefore only private solitude represents *real* solitude). Or, whether solitude is in fact an inward-focused psychological state that can exist in physical aloneness or in the presence of others as long as one is not interacting with others.

The current study sought laypeople’s views on the nature of solitude to define it. In past research, laypeople have been similarly tapped to expand on intuitable (e.g., because they are in the public discourse or accessible human experiences) ideas that are scientifically examined, and they can offer a broader and multifaceted perspective to the scientific discourse. For example, laypeople have been queried about how body mass index (BMI) relates to their understanding of ideal body weight (Crawford & Campbell, 1999); to grasp the nature of religion and spirituality (Schlehofer et al., 2008); and to characterize mindfulness (Haddock et al., 2022). In some contexts, such as when defining quality of life, lay responses have been used to create taxonomies that describe constructs through their multiple facets (Farquhar, 1995). In others, researchers have queried laypeople to inform scientific discourse when scientific definitions are too narrow (e.g., in the case of healthy aging, Hung et al., 2010). Indeed, in the health sector it has been argued that rather than being watered-down versions of scientific definitions, popular perceptions are complex integrations of historical and cultural beliefs and experiences that inform and enrich scientific understanding (Hughner & Kleine, 2004).

The current article is not the first attempt to build definitions of solitude from laypeople’s perspectives. The very understanding of solitude as a distinct construct has come from researchers trying to understand whether individuals can distinguish the state. For example, Galanaki (2004) explored with children the differentiation between being alone and the feeling of loneliness and found they could recognize aloneness as an orthogonal state worth pursuing. Similar findings were reported by Buchholz and Catton (1999) stemming from their interviews with adolescents. These qualitative investigations with youngsters have since received empirical support from quantitative tests of loneliness and attitude toward solitude measures (Goossens et al., 2009).

More recently, in a phenomenological study conducted to understand what people do when they are in positive solitude, Ost Mor and colleagues (2020) asked participants across much of the adult age range (from 18 to 85+ years) to write about the most important aspects of their positive solitude. The authors identified categories of solitude characterized by actions or goals pursued in those moments, including quietness, spirituality, stress control, nature connection, and recreational activities. The authors also highlighted that positive solitude experiences are characterized by choice, are uniquely agreeable, and hold meaning for individuals. From those themes, they offered a description of positive solitude (PS) as:

The choice to dedicate time to a meaningful, enjoyable activity or experience conducted by oneself. This activity/experience might be spiritual, functional, recreational or of any chosen type, and might take place with or without the presence of others. It is independent of any external or physical conditions; yet, individuals have each their own setting for engaging in PS. (Ost Mor et al., 2020, p. 15)

This comprehensive description provided ample aspects of the prototypical positive solitude, including conditions (i.e., antecedents) and consequences (e.g., quality of life)—a prototype illustrating the very best aspects of solitude.

This work has catalyzed researchers’ understanding of solitude as a distinct state. However, there is still little consensus in the field about what the core definition(s) of solitude entails. The efforts to define “positive solitude” are a reaction to its alternative—loneliness—an omnibus construct that is fundamentally distinct, and often diametrically opposed, to solitude. Namely, solitude is distinguished from a closely related concept—social isolation—in part because the latter involves unwanted but unavoidable time spent alone (Chappell & Badger, 1989; Wichers, 2014), whereas the construct “positive solitude” must be self-selected and is often pursued (Ost Mor et al., 2020). As a result, recent research has made huge advances in achieving their stated aims of differentiating solitude from isolation and loneliness.

But, in building a separate study of positive solitude, affordances (i.e., positive outcomes) of solitude often have been lumped together with aspects of its core definition. While it is important that we understand the affordances of solitude, we believe more foundational work is needed to understand the basic qualities that define solitude, which can be used by researchers to guide future empirical models of how time spent alone is impacted by culture, personality, or context (i.e., antecedents) and how it yields both positive and negative experiences.

In part, we still lack an understanding of whether, or how, the meaning of solitude may depend upon our position vis-à-vis others. Researchers have suggested that the fundamental nature of solitude and how it is experienced relies on establishing a balance between social time and periods of solitude which connect an individual with themself (R. J. Coplan et al., 2019; Weinstein et al., in press). The distinction between social time and solitary time becomes even more unclear as we grapple with the role that technological advances play, making parasocial interactions easily available even when people are physically alone (S. W. Campbell & Ross, 2022). The increasing accessibility of remote communications and the pervasiveness of social media makes it all the more difficult to define solitude.

### Current Research

The current research sought to fill a gap in our understanding of what is central, rather than subsequent, to the definition of solitude, and what psychological processes and environmental affordances precede and give rise to solitude. Based on a phenomenological epistemology (see review by Davidsen, 2013), we designed the current study to explore the subjective experiences of solitude in everyday life by considering how individuals from diverse backgrounds and adult ages understand its meaning and how they view the balance between solitude and social time. Said another way, expert researchers may endorse a conceptualization of solitude as someone physically alone or not interacting with another person which allows them to operationalize it and study it objectively (i.e., positivistic perspective), but this research was designed to understand how the word “solitude” can carry different and subjective meanings for people (i.e., phenomenological perspective), taking a bottom-up approach that relied on participants’ first-person accounts to build a better understanding of what “solitude” entails.

To understand the meaning of solitude derived from subjective experiences, there were several phenomenological approaches we could take. One popular approach is interpretative phenomenological analysis. This approach is commonly used in clinical research to study in-depth stories of a relatively homogeneous group of research participants that have gone through a similar experience, such as a chronic illness or a specific type of treatment (Larkin & Thompson, 2012). However, we did not aim to highlight the uniqueness of individual cases of solitude; instead, we wanted to survey a broad range of experiences. Reflexive thematic analysis was more appropriate for this purpose because we integrated perspectives across a heterogeneous group (Braun & Clarke, 2021).

We sought to understand solitude by considering how individuals position themselves with respect to others (paradoxically, what role do others play in our solitude?) in two ways:

**Research Question 1:** In defining solitude for ourselves, does it matter if we are with/without people?
**Research Question 2:** When we are on our own, are we longing to be with others or trying to avoid them? Or are we looking to solitude to balance what we get from our core selves with what we need from others?

## Method

### Participants

We conducted 60 in-depth interviews with adults aged 19 to 80 years who were recruited through advertisements within the community and Prolific Academic. We sought to maximize participants in terms of culture, education and socioeconomic level, gender, adult age, and geographic location. Therefore, we intentionally recruited participants from a cross-section of backgrounds by using a stratified sampling approach for 75% of the sample (*n* = 45) using Prolific Academic’s prescreen questions regarding ethnicity, country of origin, age, and gender. Participants were from 20 different countries of origin and represented individuals of various ethnicities. All were conversant in English, the language in which interviews were conducted. The interviewer experienced only occasional difficulties in terms of language and, in those cases, the interviewer would ask for clarification or paraphrase what the participant had said and ask if she had accurately represented the participants’ meaning. See the project website (osf.io/xpj37/) for a table listing participant characteristics. Ten standard questions, and follow-up inquiries, were asked of all participants. The questions were designed and ordered to allow participants to describe what alone time looks like for them and what meaning it has—or not—to well-being (the latter for a separate project). Data were extracted from anywhere within the interview when relevant for understanding the nature of solitude. Data feeding into themes are also presented in a supplementary table on the project website.

We also sought participants who could speak in-depth about their personal experiences of solitude, including but not limited to positive solitude, by selecting 75% of the sample after they responded to our own prescreen question “what is your experience of being alone?” The first author selected those individuals who (a) reflected on their internal experience (e.g., feelings, thoughts), rather than just behavior (e.g., watching TV); (b) used full sentences to describe their experiences; and (c) demonstrated some level of self-inquiry about their experiences (e.g., I felt X because of Y; I would have liked to have been. . .). This approach was taken because we were also investigating resilience predictors at personality and situation levels for a separate project (Hansen et al., under review) and felt that surface descriptions of solitude could not take us much past the existing body of work.

As a result of these procedures, our participants were likely biased toward understanding solitude in a generally positive way and ensuing definitions they provided are especially applicable to positive solitude. However, beyond selecting participants who *could* reflect deeply about experiences of solitude, we did not orient participants toward positive solitude or exclusively to positive experiences within solitude. Instead, all questions referred to solitude, broadly (e.g., “What comes to mind when you hear the word “solitude?” What does *solitude* mean to you, personally?”).

Interviews took place by videoconference or phone and lasted approximately 45 to 60 min. Interviews were semi-structured, and started with a question to directly address our inquiry here: What does solitude mean to you? The interview schedule can be found on osf.io/xpj37/, and the planned analysis process can be found on osf.io/upmh5. This registration lays out logic and plans before data collection and again at the start of analyses. As data were analyzed through reflexive thematic coding, we did not use a codebook. However, we recorded our thoughts at the start of the study and early themes for some understanding of how our own thinking evolved on the project. The final interview schedule was produced after additional conversations between the research team members.

### Meaning of Solitude

Interviews began with the question, “What comes to mind when you hear the word ‘solitude’?” The decision to do this was made for two reasons. First, solitude often has a negative connotation (outside of the psychology literature, dictionary definitions conflate it with being “alone,” “lonely,” or “uninhabited”), and that gave respondents an opportunity to distinguish between the outside perception of solitude and how they personally experience it. Second, solitude is in the eye of the beholder. Our dozens of lengthy, qualitative interviews confirmed that one’s experience of aloneness is as simple or complex, as freeing or confining, as our own perceptions and circumstances. We got informative and inspirational takes on what it means to them, personally, and how that may differ from the conventional definition. By talking about participants’ activities in solitude, and what is desirable about that state, we were able to reflect on how those needs and desires shaped a person’s definition of solitude and understanding of their solitude space.

### Positionality Statement

The research process benefits from a reflection on the social and political perspective of the authors because, inevitably, questions are designed and findings are processed through the lens of their worldviews (e.g., Rowe, 2014). As researchers, we are aware that everyone moves through the world with bias. We have preconceptions about others, just as they categorize us based on what they see and hear. But we also know that making assumptions is fruitless because it dismisses the tremendous variety of human experiences that influence our worldviews. Setting aside preconceptions entirely is impossible but being aware of them during the course of research and investigation and striving to be conscious and continuously self-reflective while actively seeking alternative ways of seeing can allow for evolving viewpoints and can guide robust analysis. The authors developed a social identity map that explores their sociocultural qualities and the impact this may have had on their thinking (Jacobson & Mustafa, 2019).

This team of authors includes three researchers raised in different countries and with different languages, religions, and socioeconomic backgrounds (see osf.io/xpj37/). We do not claim to be able to sidestep prejudice, but with decades of combined experience in conducting interviews we have learned to let other people tell us who they are (and not assume we know based on demographics). Our objective is to stay aware of potential influences, display openness and genuine inquisitiveness, and to practice a lack of judgment, both to encourage subjects’ candidness while also yielding the most accurate and complete answers to research questions.

### Data Analysis

Data were analyzed using reflexive thematic analysis (Braun & Clarke, 2006, 2012), which allowed a detailed yet systematic accounting of data based on patterns we freely observed within the data (Braun & Clarke, 2006). The second author identified an initial list of codes informed fully by the data, and the second and first authors discussed conceptual models that best described the relationships between those codes. The third author was not involved in the initial coding procedures but contributed to further refining the conceptualization after an initial model had been formed. The first and third authors are familiar with the literature on solitude. They aimed to be open while recognizing such knowledge would bias their understanding of the data. We used a multistep process to code data, starting with familiarizing ourselves with data at the outset and then generating initial and, ultimately, increasingly refined codes. We created themes keeping in mind their internal homogeneity and external heterogeneity (Braun & Clarke, 2006; Patton, 2005). Multiple steps were taken to increase the rigor of the research, including considerations of data saturation and bias. The first of these, data saturation is understood in terms of building rich data that expands the scope and replication of the study (Morse, 2015). This was achieved by recruiting strategically for diversity, asking complementary interview questions that attempted to elicit views on the topic from different angles, and examining the saturation of responses along different aspects of the coding scheme throughout the process of collecting data. We also considered reflexivity, the perspective, and background of the lead interviewer and analyst (Schoenberg et al., 2007). The lead interviewer kept a diary of interviews, codes, and her assumptions and reactions: (osf.io/xpj37/) following best practices (Ezzy, 2002).

## Results

### Context Observed

Some participants spoke about how they have experienced both negative and positive periods of solitude across their lifetimes, although the vast majority described presently having a positive relationship with time alone. Many even actively anticipate, plan for, or seek periods of solitude as a way to strike a balance with their noisy, outer worlds. By first asking participants in our qualitative interviews what came to mind when they heard the word “solitude,” we allowed participants to explore their personal meaning for time alone. We also heard about a wide variety of circumstances in which people tap into the benefits of solitude, as a time and space in which to, for example, relax, meditate, self-reflect, or pursue a goal.

Interviews took place in 2020–2021 and so inevitably touched on experiences during COVID pandemic isolations. At the start of the discussion, the interviewer recognized the change in lifestyle as the start of the pandemic and gently encouraged participants to think about solitude across time (recently and before the pandemic). Indeed, some responses referred to moments of pandemic-related solitude, although most others focused more broadly on solitude memories across time. Thus, the following themes can be thought of as colored by COVID experiences but not restricted to them.

### Meaning of Solitude

#### Overview of results

**Figure 1.**
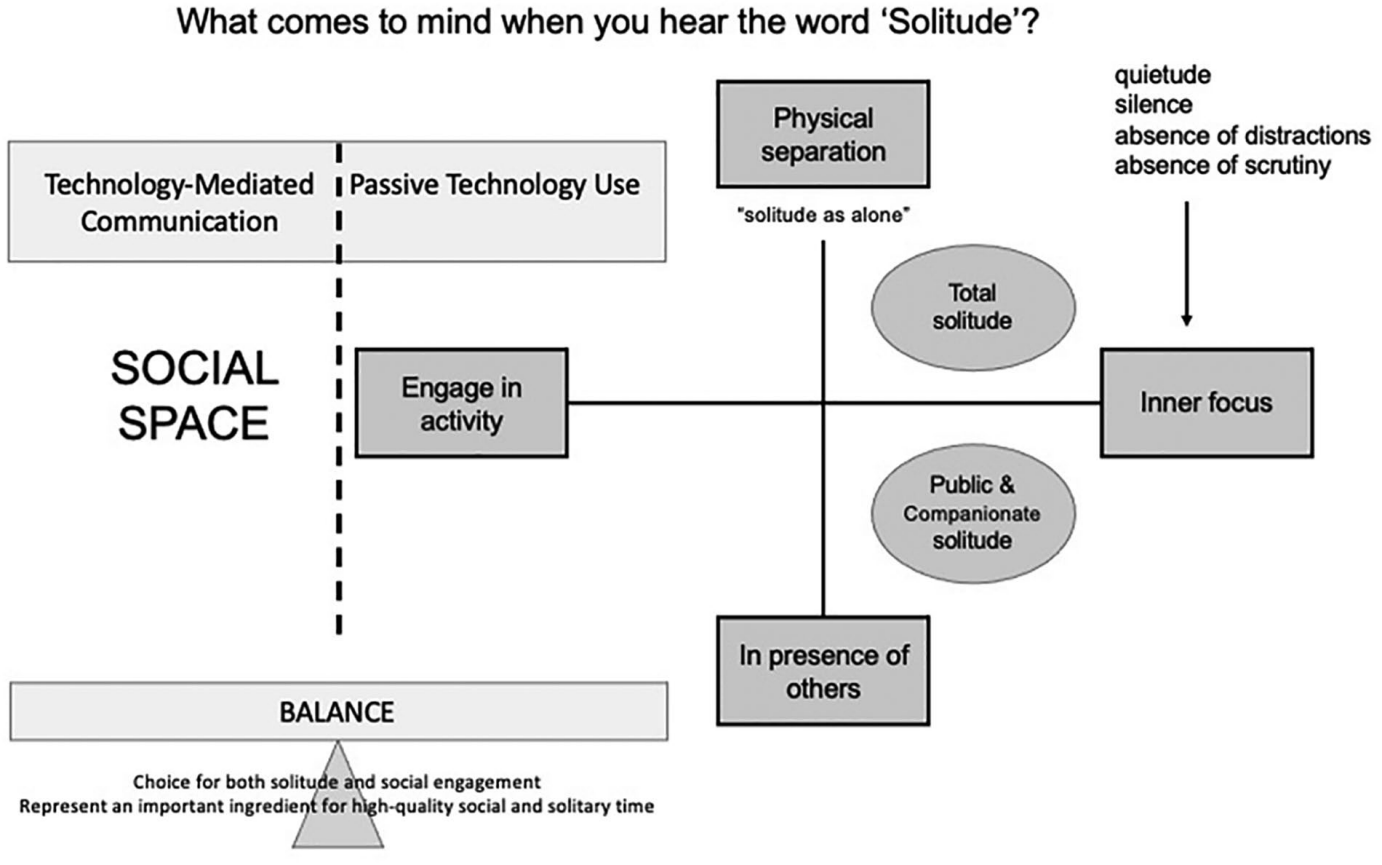
Conceptual model reflecting themes.

Solitude has a different meaning to each individual. In fact, it seems, we all have our own definition, and our own set of conditions or expectations that makes alone time a positive experience. That said, in our qualitative interviews, we identified several basic themes, summarized in Figure 1, relating to how people define moments of solitude in their daily lives. These themes surround whether or not a person needs to be physically alone for solitude to be gained, and whether or not they need to be mentally, or psychologically, apart from others. While physical separation was important to most people, there was no consensus around needing it to achieve solitude. There was, however, a strong consensus that solitude requires a mental separation from others. As a result, our conceptual model recognizes both types of separation (physical and mental) as legitimate forms of solitude. We identified the following subsections of themes (listed as subheadings below), which were most central to definitions of solitude. Examples of each theme can be found in Table 1.

**Table 1.** Participant Quotes (Right Column) Reflecting Themes Across Categories (Left Column).

Theme	Quote (participant ID)
Physical separation	Even trying to find solitude in the house if other people are here, it’s not the same for me, I feel quite twitchy about it. So, I think it’s about having enough solitude. (P02)
	Because obviously when you’re alone in solitude time you’re always in a private, mainly in a private space or in your own kind of comfortable space, so what are the impacts of being in a public space, that would be quite interesting to understanding this as well. (P43)
	Solitude, to me, means being completely on your own with nobody else around, at all. You know, maybe—it could be anywhere. In your own home, in an open space. You know, really, it could be anywhere. (P26)
Public solitude	If I’m given a long train journey, that’s brilliant for me, so long as nobody sits next to me, because I can just look out of the window and think. (P02)
	I do believe solitude helps, yeah, helps oneself to know themselves better, know their strengths and weaknesses, just helps people to know another side to themselves that they might not have known before. It’s, yeah, I enjoy sometimes just being—just taking a walk to the park alone, just reflecting. Solitude gives people chance to self-reflect, self-growth, and self-learning. . .As well as, eventually, it helps to develop self-confidence. (P50)
Companionate solitude	I just have these vivid memories of long walks, my father was very into fishing and we would often go with him and just sitting at the riverbank dangling my feet in the River Thames, just next to my father in perfect peace and quietness. That’s a very, very vivid memory, just sitting there perfectly quiet, not doing anything, just sitting next to my father perfectly quiet, not because I had to but I enjoyed it. (P36)
	It doesn’t have to be completely on my own physically; it may be in nature, it may not be; it might be sitting by the fire in the living room. It might be companionable silence with somebody. (P05)
Total solitude	It’s good for me to have my own personal space, both physically and mentally, because that’s what I’m used to having had. (P30)
	You begin to understand the relationship between your own kind of internal feelings and emotions as reactions to life compared to what happens on the outside. So, that kind of internal reflection can give you time to breathe and pause, and give myself the time that I deserve compared to always reacting to externalities. (P43)
	Even when you’re just on your own, you can go quite deep to kind of where your mind goes and how it sort of tries to process everything that you’ve encountered. (P59)
Solitude requires quiet, or silence	Peace, quiet, on your own, like you’re fishing, nobody else around, lovely, lovely river, lovely location, fishing away. Peace, quiet, babble of a brook maybe. (P22)
	I often enjoy the silence. Yeah, just enjoy the peace and tranquility. Yeah, just enjoy that. And being able to get on with something without interruption. (P26)
Solitude is diminished by distractions	I don’t have to feed anybody or take them anywhere, or answer the doorbell or whatever. . .being alone and sort of being in control of what gets done, when and how, oh, that’s lovely. (P09)
	I quite welcome sometimes the peace to be alone, to switch off from the outside world and just be me, and not have to worry about anything or anyone. (P36)
	It’s the fact that when you’re alone you don’t have any—you’re not being watched by anyone. You don’t have anyone to worry about. (P40)
	I just feel like when you’re alone and you’re in a solitary space, there is no audience, in a way . . . there is nobody to kind of present to, or in a way, engage with that energy, or conform to that energy that they’re giving. (P49)
	Solitude for me is like I’m totally disconnected from other people . . . it’s just stuff by myself, and my own thoughts for better or for worse. In my case, I tend to think that’s better. (P47)
Solitude is facilitated by chosen activities	I love long drives. I love to just go and drive alone with good music typically. I blast my music, I sing along, I come back hoarse. (P13)
	I write, and that’s a big part of solitude for me because it means I can actually think and clear my head. (P02)
	When I’m reading there’s really only one thing I’m looking [at]. As much as I’m thinking about other things it’s very focused and I don’t get a pop up and I don’t get a ring, and I don’t get a whatever . . . it’s also very, it’s just very calming. It’s a very relaxing process for me. (P17)
The role of balance in defining solitude	I’ve learnt that I need this downtime, quiet time, solitude where I can just be. It’s different from being home and feeling completely on your own. It’s a very different feeling . . . I hadn’t quite realised how important it was and how it maybe is quite an important, a very important part of my life to maintain equilibrium actually. (P01)
	I guess it was more just seeing the contrast in busy social situations and school, and then just me seeing that, oh, actually, this is really good to just have time to think by yourself without anyone around. (P08)
	You can have solitude whilst still having a sense of connectedness, and I think exploring those ways and giving people opportunities to tap into that dynamic is really valuable, because life is a flow in and out of it really in my experience. (P35)
Me time	And then so you’re just like, if I want to be happy right now, I could be happy. If I want to be sad the next second, I can be. It’s just like whatever I want to do. It’s me time. It’s like whatever energy I want to give, whatever emotions I want to feel, whatever thing I want to present right now, it’s me. (P49)
	So, it’s “me time” in the sense that, yeah, it’s just me and the dog for two days. So, I can do things without fear of being told off and what have you. But, at the same time, it brings us closer together because I’ll be anticipating preparing a nice meal for when she comes home. (P24)
Balance underlines inner focus	You begin to understand the relationship between your own kind of internal feelings and emotions as reactions to life compared to what happens on the outside. So, that kind of internal reflection can give you time to breathe and pause, and give myself the time that I deserve compared to always reacting to externalities. (P43)
	I can have that balance so I have those quiet nights to myself where I can do my study, and I can do my reading and I can reflect and do what I need for me. (P56)
Achieving balance requires choice	I guess, I’ve been lucky to have that balance that a lot of people never get quiet time or never had their own room to reflect on stuff. (P08)
	I would say it’s not always meaningful, but it always has the potential to be meaningful, and I think the deliberateness with which you step into it can make it so if you want it to. (P35)
	Well, I think we need a balance. I think the thing that makes—aside from the fact that you need to be comfortable in your own skin and your own mind and heart, you could sit with yourself, you know—I think choice again. You’ve got a choice. (P06)
Extroverts needs balance	Sometimes I feel maybe, as much as I’m a social person, the time alone with myself, and alone with my thoughts are still very welcome. (P49)
	I consider myself the kind of person who needs me time. So, I really enjoy time with my friends and doing something together but then I just need to gather some thoughts, think about my experiences . . . to break things apart and just figure it out. (P39)
Communicative forms of technology	So I think for me I would think solitude refers to being able to be myself without the social media or anything else. (P23)
	I’m like, am I ever actually in solitude, with all the things bombarding us, with social media, all of those things? (P47)
	We’re on the internet, or we’re on our cell phones. And when you look at that, then you kind of compare yourself to others being like, “Oh, wow, look at this other model, or these other gay people.” (P49)
	We have a lot of things that can comfort us. We have Skype, we have Zoom, we have Facebook, we are always—technically we’re not alone, technically. Physically we’re alone but we can always talk to somebody. (P38)
	Especially with social media and that, you’re aware that there is a world out there. So possibly that means you may not necessarily feel alone. (P60)
	[I]. . .think it’s just nice to be a friendly voice on the end of a phone or something, just to say hi and check in on them. (P56)
	I would Skype and Zoom, and Messenger with the important people in my life so it wasn’t like I didn’t see anyone or talk to anyone (P13)
Passive forms: negative	So you’re on Instagram, you’re on Facebook, you see what everyone else around you are doing, and kind of would be jealous. (P46)
	I think it’s easy to still get sucked into, like, the furnace, the Facebook and the endless scrolling on apps designed to keep your brain scrolling down. I think that is always the most frustrating bit for me, if I notice that I’ve been doing that too much, because it’s very much something I don’t want to do and when I slide too much in that then it does feel like wasted time, non-meaningful time. (P35)
Passive forms: positive	Part of the time I’m scrolling Facebook or Instagram, like it’s more of like I want to see other people and what they do, what they post, what they like. (P57)
	I mean the other thing I have to say about the current time is that sometimes I’ll choose to go to a pub or to a café on my own. I suppose, again, because I’m in control of what I do there. I don’t have to talk to other people, I can sit and read, I can look at Twitter, whatever it may be, and in fact the new technology and communicative technology has probably made me even more motivated to go and spend time on my own because I can interact at a distance as much as I want to, but no more than I want to. (P20)
Passive technology complements solitude	I would just be spending the weekend in my room and I would have the curtains drawn and put the TV on and I would just consider that as time by myself because I didn’t really see anyone else in the house because everyone’s just in their room doing their own thing. (P54)
	And when I think about that, me just on my computer, YouTube, looking at silly things, and just really enjoying my time alone. (P49)
	I’m quite happy just to sit, put some music on, put the TV on and I can entertain myself for actual days without needing any human contact at all. (P55)
	If I want to relax I go to my living room, I relax with a book, I watch TV. (P38)

#### Solitude involving physical separation

For the clear majority of our participants, physical separateness from others was an important factor in achieving positive solitude. It matters if others are around because it affects one’s ability to settle into a meaningful space in time alone (e.g., writing, cooking, meditating, dancing, and singing). In many interviews, we heard words and phrases like “solitary space” and “completely shut off” to describe how and when participants are able to access positive solitude. As solitude was defined by some totally in terms of physical separation from others, we labeled this physical separation as a recognizable form of solitude titled, simply, *being alone.*

Solitude, to me, means being completely on your own with nobody else around, at all. You know, maybe—it could be anywhere. In your own home, in an open space. You know, really, it could be anywhere. (P26; see additional examples in Table 1)

#### Solitude is a mental space characterized by inner focus

For many people, solitude means being in a space where they can focus solely on, and connect exclusively with, themselves. These internal experiences were characterized by an inner focus, for example, by attending to one’s “inner world,” thoughts, or feelings or generally connecting to oneself. Or in the words of one of our participants, “In the mental world, oh, you’re free to do virtually anything you want—just float off—you can be Superman if you want.” (P22) We noted that for many, establishing inner focus was more pressing in defining solitude than strict physical separation, and we identified two subthemes that characterized that state of mental separation in the presence of others: public solitude and companionate solitude.

##### Public solitude

Participants described achieving positive solitude with “a lack of human engagement,” and by creating a defined mental distance from others, even in a place crowded with people while walking in a park, riding on a bus, reading in a cafe, or swimming in a pond. Even at those times, they feel they are still autonomous agents because they are psychologically alone, provided they remain disengaged from others.

It’s, yeah, I enjoy sometimes just being—just taking a walk to the park alone, just reflecting. (P50)

##### Companionate solitude

For a subset of participants who did not describe physical aloneness as essential to their definition of solitude, the emphasis was to feel, or perceive, themselves as mentally distanced from others, even if the physical distance was not desired, or achievable, in practice. This could be thought of as “companionable silence” in the context of a long-term partnership in which two people are sitting in a room, together but pursuing their own thoughts and interests, or even while traveling with a close friend and having different personal perceptions and experiences of a space. Some participants described this state as creating their own “bubble” in which they are technically with someone else but are “alone in my head” and feel free to pursue their own interests.

It doesn’t have to be completely on my own physically; it may be in nature, it may not be; it might be sitting by the fire in the living room. It might be companionable silence with somebody. (P05)

##### Total solitude

For some participants, the two experiences—physical separation and internal focus—were often mentioned together as if they were simultaneous requirements of solitude. To represent this state of both (and all) aspects of solitude and consistent with recent solitude work (Nguyen et al., 2021; there called “true” solitude), we termed this *total solitude*.^1^ Extrapolating from the themes explored below, we posit that “total” solitude has both physical and mental separation, representing the most conservative meaning of solitude described by participants.

Even when you’re just on your own, you can go quite deep to kind of where your mind goes and how it sort of tries to process everything that you’ve encountered. (P59)

### Drivers of Inner Focus in Solitude

While our focus was on definitions of solitude as described above, we noted the presence of what seem to be proximal drivers of solitude. Specifically, participants described several overarching themes that seemed to promote the mental (not physical) aspect of solitude. At first glance, they appear contradictory, but the diversity of conditions is consistent with the complexity of each individual defining solitude in their own way.

#### Solitude can benefit from quiet

For a few people, positive solitude means ultimate quiet—sitting in a silent room while drawing or enjoying the silence of swimming underwater. For many, silence was required to achieve internal focus and, therefore, meaningful solitude.

#### Solitude is diminished by distractions

The reasons why physical aloneness is critical varied among participants, but they followed some common themes around being purposefully “disconnected” from others, including entering a desirable space lacking stimulation or distractions, including other people’s opinions.

For many participants, being in positive solitude required physical separation from others because the space represents a freedom from needs and desires other than their own. Physical separation opens up an opportunity to focus on one’s own needs and desires. Physical separation was also important to many because the absence of others also means a lack of perceived attention or scrutiny from others. For many of our interviewees, physical separateness was the starting point for them to enter a space in which inner focus is possible and productive. This was true particularly for parents or caregivers living with dependents and for people whose work requires caregiving and sustained empathy.

I quite welcome sometimes the peace to be alone, to switch off from the outside world and just be me, and not have to worry about anything or anyone. (P36)

#### Solitude is facilitated by chosen activities

Others are less concerned with blocking outside stimuli, and instead, their focus is directed toward (while still being physically alone) chosen activities engaged in during alone time. To them, solitude can be achieved while driving alone in a car and singing to blaring music, or watching a favorite television series at home alone.

I love long drives. I love to just go and drive alone with good music typically. I blast my music, I sing along, I come back hoarse. (P13)

### The Role of Balance in Defining Solitude

In most of our qualitative interviews, participants defined or described solitude in terms of the extremes of either constantly being with, or without, others, and many expressed a desire to, and practice of, striking a balance between solitude and social time. Even in one extreme case in which one participant has lived, by choice and for decades, in only seasonal interaction with others there was an expressed desire to take advantage of human contact when it was available.

I’ve learnt that I need this downtime, quiet time, solitude where I can just be. It’s different from being home and feeling completely on your own. It’s a very different feeling . . . I hadn’t quite realised how important it was and how it maybe is quite an important, a very important part of my life to maintain equilibrium actually. (P01)

Interestingly, comments on balance were made unprompted; there was no specific question posed about a need or desire for “enough” time alone or together with others. Participants were aware of various needs and obligations related to both states and sought to honor those according to their individual needs, whenever possible. The topic of balance was raised in several cases by participants who alluded to their definition of solitude as “me time.” That concept, of choosing time alone as an act of self-care, was positioned in contrast to time with friends or family. “Me time” was spent entertaining one’s own thoughts and emotions and pursuing one’s own interests.

And then so you’re just like, if I want to be happy right now, I could be happy. If I want to be sad the next second, I can be. It’s just like whatever I want to do. It’s me time. It’s like whatever energy I want to give, whatever emotions I want to feel, whatever thing I want to present right now, it’s me. (P49)

#### Balance underlines inner focus

Participants described the right balance as leading to solitude that is productive and focused internally—solitude that is rejuvenating. In those circumstances, solitude is not characterized by what it lacks—they are not missing interactions with other people. Instead, they are tending to their own needs and desires—and that emphasis on inner focus facilitates solitude being a useful place of self-reflection and, often, growth.

I can have that balance so I have those quiet nights to myself where I can do my study, and I can do my reading and I can reflect and do what I need for me. (P56)

#### Achieving balance requires choice

Achieving a balance between the solo and the social requires the ability to see the need for that balance and the latitude to be able to choose when to engage in one or the other. Some of our participants described having to carve out time for themselves in their daily routines, and even demand it in some cases, understanding the personal importance of balance. Several also expressed the notion that being able to spend time in solitude was an opportunity, an advantage, and even a privilege of which they gladly partake.

I guess, I’ve been lucky to have that balance that a lot of people never get quiet time or never had their own room to reflect on stuff. (P08)

#### Extroverts needs balance

Balance plays a role in the meaning of solitude, even for extroverts. Some people who described themselves as extroverts or “the life of the party”—who understood themselves to be recharged in interactions with others—also felt the need to step away from that public persona to engage solely with their own thoughts. Others who thrive in relation to family and friends also described carving out time for themselves in solitude.

### The Role of Technology in Defining Solitude

Communicative forms of technology (e.g., phone, social media) where individuals were actively conversing with another through their devices were seen to reflect brief breaks in solitude. Some participants saw them as counter-definitional:

So I think for me I would think solitude refers to being able to be myself without the social media or anything else. (P23)

The benefit of these communicative forms of technology was that they could be used strategically to enable participants to move in and out of solitude to social spaces at will, a benefit that spoke to earlier themes of having a balance between the two forms of experience.

Especially with social media and that, you’re aware that there is a world out there. So possibly that means you may not necessarily feel alone. (P60)

Passive forms of social media were not antithetical to solitude but rather created a proxy social world that could be observed by participants. For some, the salience detracted from *positive* experiences of solitude by cuing people into their social worlds. But for others it was quite beneficial. Thus, passive forms of social media did not define positive or negative solitude but were one of many activities that could be engaged in during solitude. In all, passive technology complemented solitude, especially for “being alone” and unwinding. Our participants described their solitude time involving passive television watching, listening to music, or using the internet for reading and researching. These activities were part of their relaxation or diversion methods, and were seen as neutral or even beneficial aspects of solitude.

And when I think about that, me just on my computer, YouTube, looking at silly things, and just really enjoying my time alone. (P49)

## Discussion

To explore the defining characteristics of solitude, we asked adults to explore its meaning from their own perspectives. We were particularly interested in learning whether solitude is a state that specifically requires physical aloneness or if a state of psychological aloneness can be reached without physical separation from others. There was little consensus among participants about whether solitude was either totally a physical or a mental state. For some, physical separation was key, but many others felt strongly that they could have solitude even in the presence of close others by looking inward. Our findings suggested that a retreat from immediate social demands, and noncommunication in general, form the basis of solitude and involve either mental separation or physical separation. This core definition supports Long and Averill’s (2003) conceptualization of solitude as, “a state characterized by disengagement from the immediate demands of other people” (p. 23), and S. W. Campbell and Ross’s (2022) definition of solitude as noncommunication. Disengagement may mean a complete absence of other people, or it may exist on a continuum defined by levels of inner focus, and levels of external distraction or demand.

There were, therefore, multiple expressions of how individuals perceived the state of solitude, and the factors that facilitate or foil beneficial solitude. Several participants set an early alarm to get a few minutes to themselves in the morning before a would-be noisy home stirs to life. For them, solitude requires total aloneness and quiet. Others defined solitude as mental distancing from others that are nearby. For example, in a public space when other people are around, solitude was characterized by a withdrawal of attention from others, an escape from one’s social surroundings, a look inwards, or a sensation of peaceful calm. Some felt they were in positive solitude alongside another in “companionable silence” or could tap into solitude happily on a busy, city bus.

The data suggested that it is most appropriate to recognize a taxonomy of solitude experiences, each with its own distinct characteristics. Considering participant descriptions as a whole, we conceived of a two-dimensional conceptual structure for defining solitude that resonates with the previous conceptual and operational approaches taken in the study of solitude (e.g., Long et al., 2003; Nguyen et al., 2021). Building knowledge in the field requires researchers to recognize how they are defining solitude, and how that definition relates to the observed effects of their study. Those parameters also allow researchers to consider how their findings may relate to other forms of solitude and may indicate promising paths for future research.

### A Taxonomy of Solitude

Nguyen et al. (2021) proposed conceptual distinctions between *true* solitude (in this article, we call it “total” solitude to avoid implying other kinds are *untrue*) and other types of solitude that include either presence of others (i.e., public or companionate solitude) or engagement in chosen tasks and activities. We represented those conceptual distinctions in the model depicted in Figure 1. Participants were consistent in views that solitude is qualified by both physical separation and mental distancing from others. In this sense, the ways our participants thought about the word “solitude” are consistent with how previous literature has operationalized this state (e.g., physical separation is consistent with Larson, 1990; mental distancing is consistent with Long & Averill, 2003). However, it seems from our current findings that *any* time spent physically separated from others can constitute solitude, regardless of whether someone chooses to do nothing or something. Such “alone” time may include watching television, playing video games, reading a book, or working alone. Many participants described being task-focused but also having a sense of self-connection and recuperation from time with others. While we often think of solitude as time that is free from disruptions and distractions and therefore allows unadulterated inner focus, in our research, distracted alone time still constituted solitude for many participants. In short, the pursuit of nothingness is not necessary for solitude. Termed “solitude as alone” here, physical separation from others devoid of a clear internal focus has also been recognized as beneficial time within the existing literature focused on leisure time (Buchholz, 1999). In diary studies, “solitude as alone” has been operationalized when participants reported no one else around (Larson et al., 1982); here, whether or not participants engage in activities was not an important qualifier. People appreciate even outward-focused solitude spent doing leisure-time activities that help them unwind. For example, the 2016 “rest test” polled 18,000 people and found that people’s favorite way to rest was through reading (BBC, 2016), and the use of technology to relax and relieve stress (Leung, 2015).

In our deeper examination of the role of technology specifically, we observed themes of communicative forms of technology being antithetical to solitude, the use of communicative (social) technology as a way of organically moving in and out of solitude, and observations that some forms of technology (including observing content on social media) may be used in solitude. For some, the salience of other people was a negative experience that interfered with well-being. However, in the case of completely passive forms of technology (such as TV and YouTube), activities were generally seen as compatible with solitude and were positive for many. We interpreted this to mean that technology only changes the meaning of solitude for our participants when it involves reciprocal communications with another person, a view that closely aligns with S. W. Campbell and Ross’s (2022) view of technology-mediated communication as undermining solitude.

Despite the salience of physical separation, the majority of participants described solitude as a predominantly or even exclusively mental space, and we identified forms of solitude that took place with others physically present. The first, “companionate solitude,” was described as an internal, private, mental focus in the company of a close other. This form of solitude was described as having a certain sense of comfort that came from partners (often, but not exclusively, romantic partners) agreeing to share their internal and private time with one another. The time was described as peaceful and relaxing—an opportunity to connect with self-chosen activities that were interesting or rewarding to our participants in the company of others with whom they felt safe and comfortable.

Within the literature of solitude, this experience has been qualitatively described in interviews with individuals who go on wilderness trips with others in the same group. In a wilderness environment, the comfort with solitude shared among fellow travelers that one had not known previously can be facilitated by the grandiosity of natural surroundings (Hinds, 2011). Within the close relationships literature, some allusions to companionate solitude have been made referring to quiet time together as a value (Cantor & Malley, 2013), and in conceptual discussions of the role that silence plays in intimate relationships (Jaworski, 2011). These moments may be the result of relationships that are secure and trusting, where partners feel they can be “themselves” while together (L. Campbell & Stanton, 2019; Gable & Reis, 2006). For our participants, companionate solitude took the form of relaxing moments of shared solitude either at home or outdoors, in the presence of one close other. Extrapolating from the responses of participants, the ability to share companionate solitude with a partner may paradoxically underly intimacy and even represent a positive relationship outcome.

The second form of solitude is characterized strictly by mental inner focus. “Public solitude” consisted of being in the presence of others but with little or no connection to another in one’s immediate physical space. We distinguish this from companionate solitude because public solitude refers to situations in which participants describe being “alone” among strangers with whom the individual is not obligated or expected to interact. The lack of interaction allowed participants to turn their attention inward. There is also a unique sense of gained “anonymity,” which has been described as one of the benefits of solitude (Long et al., 2003). While this experience of anonymity has not been studied empirically in solitude literature, many sociologists have discussed the extent to which individuals can assume anonymity in shared spaces with strangers—a sense of invisibility that allows individuals to be free of social surveillance (e.g., De Backer, 2019; Hatuka & Toch, 2017; Langegger & Koester, 2016).

Although some participants described solitude as requiring physical separation or mental distancing, for others, both physical *and* mental space were necessary and interdependent to define solitude. We termed this expression “total solitude,” benefiting from both aspects of separation, which has been used as an operational definition in laboratory studies of time spent alone (called “true” solitude in past work; Nguyen et al., 2018; Pfeifer et al., 2019), where participants were left alone and without distractions in a quiet lab room for a short period of time. Arguably, it is the most conservative definition of solitude because it is clearly orthogonal to social or interactional time. Previous research has shown that total solitude helps to bring about low-arousal emotions and reduce high-arousal emotions (Nguyen et al., 2018), although it is unclear whether these regulation benefits of relaxation and a sense of inner peace extend similar to other forms of solitude. Indeed, reading for leisure, which in our categorization may entail “solitude as alone”—which may not necessarily be focused inward to one’s thoughts—has been found to bring about similar benefits (Iwasaki et al., 2005; Trenberth & Dewe, 2002). Furthermore, the evidence is mixed regarding the emotional and cognitive costs and benefits of individuals being alone with their thoughts (i.e., in total solitude), and research points to negative (Westgate et al., 2017; Wilson et al., 2014), neutral (Nguyen et al., 2021), and modifiable (e.g., capable of being improved through simple strategies that support positive thinking; Westgate et al., 2017) outcomes of time spent alone.

### Supports for Mental Separation

While physical separation was characterized straightforwardly by the presence or absence of others, mental separation seemed to require an optimal context, one that benefited from specific attributes of solitude, namely, (a) quiet or silence, (b) distraction-free, and (c) engaging in chose activities. An important support for the first of these was to experience a quiet and calm space free of distractions, consistent with both Ost Mor’s description of solitude (Ost Mor et al., 2020) and other theorizing on the qualities of positive time spent alone (R. J. Coplan & Bowker, 2013; Hall, 2001). Bridging the current and past work, choice is not essential for defining solitude and rather facilitates a positive inner focus. It is, indeed, definitional to the subcategory of solitude understood as *Positive Solitude*. Furthermore, in our work and others, it appears that solitude is linked in a meaningful way to a quiet environment that does not pull individuals in directions away from themselves. This study offered a specific relationship between quiet and solitude. In our current findings, quiet seemed a proximal *antecedent* to the “inner focus” dimension of solitude, which required the absence of noise and external stimulation, as well as the absence of distractions.

Underlying positive internal space was balance. We anticipated that balance would play a key role in solitude based on previous research (R. J. Coplan et al., 2019). While we rarely heard participants define solitude directly through “balance,” we did hear spontaneous descriptions of balance during reporting on solitude from the majority of participants. This led us to believe that balance is a contextual support that allows people to enter into the inner-focused psychological space of solitude. It may determine whether solitude plays a complementary or antagonistic role in our lives. When balanced, solitude and social activities complement one another, and solitude feels beneficial, while an unbalanced equation can feel disruptive to focus and well-being.

Therefore, in our proposed model in Figure 1, we represent balance as the bridge between social space and solitude, which are divided by a dotted line, rather than a solid one, because solitude does not mean a complete cutting off from social space. This finding builds on previous work that suggests balance may play a role in understanding solitude. Namely, evidence from farmers who spend substantial time alone suggest they actively balance their isolation with social media connections to alleviate boredom and loneliness (Vidal-González & Fernández-Piqueras, 2021). More direct early findings suggest that those who describe a balance between solitude and social time during interviews see their solitude in a positive light (Thomas, 2017).

In addition, participants described that when the “right” amounts of solitude relative to social time are chosen or selected, solitude transforms into a space that opens introspection and connection to internal experiences in a positive way. In forced or excessive solitude, the sense that one can connect or explore internal or self-processes constructively or peacefully is disrupted. It is worth noting that the “right” amount of solitude may vary, but the consensus was that nearly everyone benefited from some of this space, provided it was choiceful. Motivation, that is, whether or not people are alone by choice, was therefore key to balance. This resonates with a large body of work that highlights the importance of motivation for well-being in solitude (Nguyen et al., 2018, Thomas & Azmitia, 2019), but our participants saw that motivation is important for the perception of balance between solitude and social space. It is also worth noting that balance between solitude and social spaces was a factor even when solitude was characterized solely by mental separation (without physical separation). That suggests that as long as individuals can experience mental separation from others, physical separation may not be necessary or important.

### Future Research

The current research was designed to identify forms of solitude that elucidate the meaning of solitude to laypeople exploring their daily lived experiences, and by doing so, inform a classification of solitude types with distinct characteristics. We saw this work as providing a foundation to develop coherent bodies of future work targeting one or more forms of solitude. For example, one implicit (assumed), but not explicit (stated) theme that we identified in our study is that public and companionate forms of solitude required being able to retreat to a sheltered psychological space, with little or no immediate or pressing distractions. It would be fascinating in future research to understand what kinds of social contexts promote the ability to internally focus in the presence of others. This may involve having psychological safety (Maurer & Daukantaitė, 2020), support for authentic self-expression (Wood et al., 2008), or autonomy support (Ryan & Deci, 2006). Further research can explore how social contexts facilitate or undermine this internal focus. More broadly, the current findings suggested solitude is a space that holds psychological distance from others or an internal focus. This state may correspond with other psychological experiences, such as feeling independent versus dependent on others, or engaging in autonomous activities that are volitional and self-selected (Deci & Ryan, 2008). But it is also possible to be in solitude and feel highly dependent on others (as in the case of solitary confinement) or engage in nonautonomous activities (e.g., because of pressure to achieve a deadline). Future research can build on existing work in these areas (e.g., Haney, 2018) to understand the *interpersonal* and *intrapersonal* experiences that improve the experience of solitude but bearing in mind that these models may not hold constant across different solitude states.

In addition, it would be useful to test the costs and benefits of “total” solitude compared with solitude where others are present (companionate or public solitude) or when individuals are physically alone but not sitting with their thoughts. Some have argued that “total” solitude is also the truest form of solitude when one can attend to one’s thoughts and feelings in an unstructured space undistracted by media or other activities that demand attention (Nguyen et al., 2021). Studies regarding the emotional and cognitive costs and benefits of total solitude to individuals (Nguyen et al., 2021; Westgate et al., 2017; Wilson et al., 2014) have not yet compared this state with other forms of solitude, although direct examinations of their shared and divergent characteristics would be relevant to social psychological research, for example, studying the use of technology as a potential disruptor of solitude (Thomas et al., 2021).

Comparisons between “total” and other forms of solitude can also inform clinical research, for example, studying depression, emotion dysregulation, and rumination, areas where distractions may offer necessary relief (Wang et al., 2013). A few studies have suggested that unstructured solitude, without distractions or activities to focus one’s mind, could be problematic for people with psychological disorders. For example, a study by Larson and Johnson (1985) found that patients with bulimia engaged in binge eating more during time alone in the evening at home compared with time alone at work. Another study suggested that the incapacity to be alone explaines the association between attachment anxiety and problematic smartphone use (Bermingham et al., 2021). Nascent evidence suggested that solitary times, especially unstructured ones, could aggravate mental health problems, warranting future clinical research in this area. From this research, it may be worthwhile to examine optimal forms of solitude for individuals struggling with mental health difficulties. For example, companionate solitude may offer individuals a time to disengage but still feel a sense of connection to others.

#### Solitude as a function of culture and age

Explorations of solitude are best understood in the context of the full adult lifespan, and as contextualized within a broader culture, because each dimension may fundamentally change how it is perceived. As individuals age, they experience solitude positively and value its opportunities (Buchholz & Catton, 1999; Ost Mor et al., 2020; Weinstein et al., in press). When compared with midlife adults, older adults described solitude as a peaceful and quiet time (Ost Mor et al., 2020), and they benefit more in terms of their emotional well-being (Larson, 1990; Lay et al., 2019; Rokach & Brock, 1998). Solitude affordances may also change based on culture (Averill & Sundarajan, 2014). Culturally specific forms of extreme solitude exist, such as the *hikikomori* phenomenon in Japan (Teo et al., 2014) that is also reported in other cultures (Kato et al., 2012). Everyday solitude can also vary as a function of culture. For example, East Asian heritage predicts more positive and less negative experiences of solitude, as does experiencing immigration (Jiang et al., 2019). To understand solitude truly, we must invest in work that cuts across culture and age. The current research did that to some extent by seeking individuals across the adult ages and from different cultures, countries, and ethnicities. We did not believe we had sufficiently large samples to systematically compare these groups. Large-scale studies are needed to address this need with greater precision and power.

#### Limitations

We see several predominant limitations of this study that bear mentioning. First, the current set of interviews were conducted—not by design—during the COVID-19 pandemic, and we cannot be certain results will generalize outside of that context, including whether some forms of solitude will take precedence over others in the future (e.g., companionate solitude may be less frequent as individuals are not obliged to spend time within their households). That said, we believe the current set of findings relates to everyday experiences of solitude which we are confident will extend beyond pandemic conditions. Many of our participants lived in relatively remote areas or were interviewed during non-lockdown periods, and they shared experiences of solitude that were similar to those who may have felt COVID-19 lifestyle changes more keenly.

Second, because we were interested in contacting individuals from different backgrounds who could speak to varied and positive experiences with solitude, we sampled almost exclusively those who were capable of having a rewarding or pleasurable relationship with time spent alone. Although we did not query these individuals about “positive solitude,” per se, their definitions of solitude naturally gravitated toward positive forms of solitude. For example, none defined solitude as isolation or loneliness—although some explored those negative experiences as more peripheral experiences during time spent alone. However, it is important to note that previous literature suggests that English-speaking children, in general, begin to distinguish between solitude and loneliness in early adolescence (Galanaki, 2004). Consistent with these findings, our taxonomy concerns either benign or positive occasions of solitude by default but does not directly identify or classify negative forms of time spent alone.

Third, we are unable to draw conclusions regarding the extent to which the current set of findings generalize to broader populations. This is because we took an approach to maximizing generalization that is different from the statistical generalization often used in quantitative research (Levitt, 2021). Specifically, while statistical generalization focuses on how collected data capture variation in the population, qualitative generalization focuses on how data capture variation in the phenomenon of interest (Levitt, 2021). To capture our construct of interest (solitude) during the analytic process, we explored patterns that did not fit with observations made in previous interviews and set out to identify variations in the meanings of solitude through this process.

Finally, the aim of our research was to identify the essential and important components of solitude (i.e., mental separation) across narratives from diverse individuals. Yet, this study was designed to represent diverse views but not to inform similarities or differences among them. Because we did not delve deeply into how each participant’s life circumstance might be linked to what solitude means to them (some of our participants may have mentioned some specific conditions but we did not inquire exhaustively), findings might not be transferable to unique cases of solitude for those with extraordinary lifestyles.

## Conclusion

This work was designed to describe core definitions of solitude, and we identified key forms of solitude that have previously been described in siloed studies within the solitude literature: being alone, companionate solitude, public solitude, and total solitude. We suggest that researchers studying solitude could systematically explore one or more of these forms. It would be fascinating to understand what psychological processes and outcomes are shared between solitude forms, but we recognize that differences may exist which preclude such generalizations.

## Supplemental Material

sj-docx-1-psp-10.1177_01461672221115941 – Supplemental material for Definitions of Solitude in Everyday Life Click here for additional data file. Supplemental material, sj-docx-1-psp-10.1177_01461672221115941 for Definitions of Solitude in Everyday Life by Netta Weinstein, Heather Hansen and Thuy-vy Nguyen in Personality and Social Psychology Bulletin

## Notes

1 We selected to name this theme “total” rather than “true” solitude because it did not appear “truer” to lived experiences of solitude than other forms participants identified. Rather, it was total because it involved both critical aspects of solitude experiences—physical *and* mental separation.

## References

[bibr1-01461672221115941] Averill J. R. Sundararajan L. (2014). Experiences of solitude. In Coplan R. J. Bowker J. C. (Eds.), The handbook of solitude: Psychological perspectives on social isolation, social withdrawal, and being alone (pp. 90–108). Wiley Blackwell.

[bibr2-01461672221115941] BBC. (2016). https://www.bbc.co.uk/programmes/articles/5GF8npkXxpp4z0KBqGx2pl7/the-ten-most-restful-activities

[bibr3-01461672221115941] Bermingham L. Meehan K. B. Wong P. S. Trub L. (2021). Attachment anxiety and solitude in the age of smartphones. Psychoanalytic Psychology, 38(4), 311–318.

[bibr4-01461672221115941] Braun V. Clarke V. (2006). Using thematic analysis in psychology. Qualitative Research in Psychology, 3(2), 77–101.

[bibr5-01461672221115941] Braun V. Clarke V. (2012). Thematic analysis. In Cooper H. Camic P. M. Long D. L. Panter A. T. Rindskopf D. Sher K. J. (Eds.), APA handbook of research methods in psychology (pp. 57–71). American Psychological Association.

[bibr6-01461672221115941] Braun V. Clarke V. (2021). Can I use TA? Should I use TA? Should I not use TA? Comparing reflexive thematic analysis and other pattern-based qualitative analytic approaches. Counselling and Psychotherapy Research, 21(1), 37–47.

[bibr7-01461672221115941] Buchholz E. S. (1999). The call of solitude: Alonetime in a world of attachment. Simon and Schuster.

[bibr8-01461672221115941] Buchholz E. S. Catton R. (1999). Adolescents' perceptions of aloneness and loneliness. Adolescence, 34(133), 203–204. 10234378

[bibr9-01461672221115941] Campbell L. Stanton S. C. (2019). Adult attachment and trust in romantic relationships. Current Opinion in Psychology, 25, 148–151. 30096516 10.1016/j.copsyc.2018.08.004

[bibr10-01461672221115941] Campbell S. W. Ross M. Q. (2022). Re-conceptualizing solitude in the digital era: From “being alone” to “noncommunication”. Communication Theory, 32(3), 387–406.

[bibr11-01461672221115941] Cantor N. Malley J. (2013). Life tasks, personal needs, and close relationships. In Fletcher G. J. O. Fincham F. D. (Eds.), Cognition in close relationships (pp. 111–136). Psychology Press.

[bibr12-01461672221115941] Chappell N. L. Badger M. (1989). Social isolation and well-being. Journal of Gerontology, 44(5), S169–S176. 10.1093/geronj/44.5.s169 2768776

[bibr13-01461672221115941] Coplan R. C. Bowker J. C. Nelson E. (2021). The handbook of solitude: Psychological perspectives on social isolation, social withdrawal, and being alone (2nd ed.). Wiley-Blackwell.

[bibr14-01461672221115941] Coplan R. J. Bowker J. C. (2013). All alone: Multiple perspectives on the study of solitude. In Coplan R. J. Bowker J. C. (Eds.), The handbook of solitude: Psychological perspectives on social isolation, social withdrawal, and being alone (pp. 1–13). Wiley.

[bibr15-01461672221115941] Coplan R. J. Hipson W. E. Archbell K. A. Ooi L. L. Baldwin D. Bowker J. C. (2019). Seeking more solitude: Conceptualization, assessment, and implications of aloneliness. Personality and Individual Differences, 148, 17–26.

[bibr16-01461672221115941] Crawford D. Campbell K. (1999). Lay definitions of ideal weight and overweight. International Journal of Obesity, 23(7), 738–745. 10454108 10.1038/sj.ijo.0800922

[bibr17-01461672221115941] Davidsen A. S. (2013). Phenomenological approaches in psychology and health sciences. Qualitative Research in Psychology, 10(3), 318–339. 23606810 10.1080/14780887.2011.608466 PMC3627202

[bibr18-01461672221115941] De Backer M . (2019). Regimes of visibility: Hanging out in Brussels’ public spaces. Space and Culture, 22(3), 308–320.

[bibr19-01461672221115941] Deci E. L. Ryan R. M. (2008). Facilitating optimal motivation and psychological well-being across life’s domains. Canadian Psychology/Psychologie Canadienne, 49(1), 14–23.

[bibr20-01461672221115941] Ezzy D. (2002). Researching health, second opinion. In Germov J. (Ed.), South Melbourne (pp. 49–64). Oxford University Press.

[bibr21-01461672221115941] Farquhar M. (1995). Definitions of quality of life: A taxonomy. Journal of Advanced Nursing, 22(3), 502–508. 7499618 10.1046/j.1365-2648.1995.22030502.x

[bibr22-01461672221115941] Gable S. L. Reis H. T. (2006). Intimacy and the self: An iterative model of the self and close relationships. In Noller P. Feeney J. A. (Eds.), Close relationships: Functions, forms and processes (pp. 211–225). Psychology Press/Taylor & Francis.

[bibr23-01461672221115941] Galanaki E. (2004). Are children able to distinguish among the concepts of aloneness, loneliness, and solitude? International Journal of Behavioral Development, 28(5), 435–443.

[bibr24-01461672221115941] Goossens L. Lasgaard M. Luyckx K. Vanhalst J. Mathias S. Masy E. (2009). Loneliness and solitude in adolescence: A confirmatory factor analysis of alternative models. Personality and Individual Differences, 47(8), 890–894.

[bibr25-01461672221115941] Haddock G. Foad C. M. Thorne S. (2022). How do people conceptualize mindfulness? Royal Society Open Science, 9(3), Article 211366. 10.1098/rsos.211366 PMC8941388 35345432

[bibr26-01461672221115941] Hall T. E. (2001). Hikers’ perspectives on solitude and wilderness. International Journal of Wilderness, 7(2), 20–24.

[bibr27-01461672221115941] Haney C. (2018). The psychological effects of solitary confinement: A systematic critique. Crime and Justice, 47(1), 365–416.

[bibr28-01461672221115941] Hansen H. Weinstein N. Nguyen T.-V. (2022). Resilient in solitude: A qualitative multi-level analysis of the drivers of flourishing when alone. Manuscript under review.

[bibr29-01461672221115941] Hatuka T. Toch E. (2017). Being visible in public space: The normalisation of asymmetrical visibility. Urban Studies, 54(4), 984–998.

[bibr30-01461672221115941] Hinds J. (2011). Woodland adventure for marginalized adolescents: Environmental attitudes, identity and competence. Applied Environmental Education & Communication, 10(4), 228–237.

[bibr31-01461672221115941] Hughner R. S. Kleine S. S. (2004). Views of health in the lay sector: A compilation and review of how individuals think about health. Health, 8(4), 395–422. 15358896 10.1177/1363459304045696

[bibr32-01461672221115941] Hung L. W. Kempen G. I. J. M. De Vries N. K. (2010). Cross-cultural comparison between academic and lay views of healthy ageing: A literature review. Ageing & Society, 30(8), 1373–1391.

[bibr33-01461672221115941] Iwasaki Y. MacTavish J. MacKay K. (2005). Building on strengths and resilience: Leisure as a stress survival strategy. British Journal of Guidance & Counselling, 33(1), 81–100.

[bibr34-01461672221115941] Jacobson D. Mustafa N. (2019). Social identity map: A reflexivity tool for practicing explicit positionality in critical qualitative research. International Journal of Qualitative Methods, 18, 1–12.

[bibr35-01461672221115941] Jaworski A. (Ed.). (2011). Silence: Interdisciplinary perspectives (Vol. 10). Walter de Gruyter.

[bibr36-01461672221115941] Jiang D. Fung H. H. Lay J. C. Ashe M. C. Graf P. Hoppmann C. A. (2019). Everyday solitude, affective experiences, and well-being in old age: The role of culture versus immigration. Aging & Mental Health, 23(9), 1095–1104. 30621431 10.1080/13607863.2018.1479836

[bibr37-01461672221115941] Kato T. A. Tateno M. Shinfuku N. Fujisawa D. Teo A. R. Sartorius N. . . . Kanba S. (2012). Does the ‘hikikomori’syndrome of social withdrawal exist outside Japan? A preliminary international investigation. Social Psychiatry and Psychiatric Epidemiology, 47(7), 1061–1075. 21706238 10.1007/s00127-011-0411-7 PMC4909153

[bibr38-01461672221115941] Langegger S. Koester S. (2016). Invisible homelessness: Anonymity, exposure, and the right to the city. Urban Geography, 37(7), 1030–1048.

[bibr39-01461672221115941] Larkin M. Thompson A. (2012) Interpretative phenomenological analysis. In Thompson A. Harper D. (Eds.), Qualitative research methods in mental health and psychotherapy: A guide for students and practitioners (pp. 99–116). John Wiley.

[bibr40-01461672221115941] Larson R. W. (1990). The solitary side of life: An examination of the time people spend alone from childhood to old age. Developmental Review, 10(2), 155–183.

[bibr41-01461672221115941] Larson R. Johnson C. (1985). Bulimia: Disturbed patterns of solitude. Addictive Behaviors, 10(3), 281–290. 3866486 10.1016/0306-4603(85)90009-7

[bibr42-01461672221115941] Larson R. Csikszentmihalyi M. Graef R. (1982). Time alone in daily experience: loneliness or renewal? In Peplau L. A. Perlman D. (Eds.), Loneliness: A sourcebook of current theory, research, and therapy. New York: Wiley-Interscience.

[bibr43-01461672221115941] Lay J. C. Pauly T. Graf P. Biesanz J. C. Hoppmann C. A. (2019). By myself and liking it? Predictors of distinct types of solitude experiences in daily life. Journal of Personality, 873(3), 633–647. 10.1111/jopy.12421 30003553

[bibr44-01461672221115941] Leung L. (2015). Using tablet in solitude for stress reduction: An examination of desire for aloneness, leisure boredom, tablet activities, and location of use. Computers in Human Behavior, 48, 382–391.

[bibr45-01461672221115941] Levitt H. M. (2021). Qualitative generalization, not to the population but to the phenomenon: Reconceptualizing variation in qualitative research. Qualitative Psychology, 8(1), 95–110.

[bibr46-01461672221115941] Long C. R. Averill J. R. (2003). Solitude: An exploration of benefits of being alone. Journal for the Theory of Social Behaviour, 33(1), 21–44. 10.1111/1468-5914.00204

[bibr47-01461672221115941] Long C. R. Seburn M. Averill J. R. More T. A. (2003). Solitude experiences: Varieties, settings, and individual differences. Personality and Social Psychology Bulletin, 29(5), 578–583. 15272992 10.1177/0146167203029005003

[bibr48-01461672221115941] Maurer M. M. Daukantaitė D. (2020). Revisiting the Organismic Valuing Process Theory of personal growth: A theoretical review of Rogers and its connection to positive psychology. Frontiers in Psychology, 11, Article 1706. 10.3389/fpsyg.2020.01706 PMC7385226 32793057

[bibr49-01461672221115941] Morse J. M. (2015). Critical analysis of strategies for determining rigor in qualitative inquiry. Qualitative Health Research, 25(9), 1212–1222. 26184336 10.1177/1049732315588501

[bibr50-01461672221115941] Nguyen T. V. T. (2021). *An* investigation of the effects of social media browsing on leisure solitude in emerging adults. PsyArXiv. https://psyarxiv.com/ym4s2/

[bibr51-01461672221115941] Nguyen T. V. T. Ryan R. M. Deci E. L. (2018). Solitude as an approach to affective self-regulation. Personality and Social Psychology Bulletin, 44(1), 92–106. 29072531 10.1177/0146167217733073

[bibr52-01461672221115941] Nguyen T. V. T. Weinstein N. Ryan R. M . (2021). The possibilities of aloneness and solitude: Developing an understanding framed through the lens of human motivation and needs. In Coplan R. J. Bowker J. C. Nelsons L. J. (Eds.), The handbook of solitude: Psychological perspectives on social isolation, social withdrawal, and being alone (pp. 224–239). Wiley.

[bibr53-01461672221115941] Ost Mor S. Palgi Y. Segel-Karpas D . (2020). The definition and categories of positive solitude: Older and younger adults’ perspectives on spending time by themselves. The International Journal of Aging and Human Development, 93(4), 943–962. 32938200 10.1177/0091415020957379

[bibr54-01461672221115941] Patton M. Q. (2005). Qualitative research. John Wiley.

[bibr55-01461672221115941] Pfeifer E. Geyer N. Storch F. Wittmann M. (2019). “Just tink”—sudents feel significantly more relaxed, less aroused, and in a better mood after a period of silence alone in a room. Psych, 1(1), 343–352. 10.3390/psych1010024

[bibr56-01461672221115941] Rokach A. Brock H. (1998). Coping with loneliness. The Journal of psychology, 132(1), 107–127.

[bibr57-01461672221115941] Rowe W. E. (2014). Positionality. In Coghlan D. Brydon-Miller M. (Eds.), The SAGE encyclopedia of action research (pp. 628–631). SAGE.

[bibr58-01461672221115941] Ryan R. M. Deci E. L. (2006). Self-regulation and the problem of human autonomy: Does psychology need choice, self-determination, and will? Journal of Personality, 74(6), 1557–1586. 17083658 10.1111/j.1467-6494.2006.00420.x

[bibr59-01461672221115941] Schlehofer M. M. Omoto A. M. Adelman J. R. (2008). How do “religion” and “spirituality” differ? Lay definitions among older adults. Journal for the Scientific Study of Religion, 47(3), 411–425.

[bibr60-01461672221115941] Schoenberg N. E. Shenk D. Kart C. S. (2007). Food for thought: Nourishing the publication of qualitative research. Journal of Applied Gerontology, 26(1), 4–16.

[bibr61-01461672221115941] Storr A. (1988). Solitude: A return to the self. Free Press.

[bibr62-01461672221115941] Teo A. R. Stufflebam K. W. Kato T. A. (2014). The Intersection of culture and solitude: The hikikomori phenomenon in Japan. In Coplan R. J. Bowker J. C. (Eds.), The handbook of solitude: Psychological perspectives on social isolation, social withdrawal, and being alone (pp. 445–460). Wiley. 10.1002/9781118427378.ch25

[bibr63-01461672221115941] Thomas V. D. (2017). How to be alone: An investigation of solitude skills. University of California, Santa Cruz.

[bibr64-01461672221115941] Thomas V. D. Azmitia M. (2019). Motivation matters: Development and validation of the motivation for solitude scale–short form (MSS-SF). Journal of Adolescence, 70, 33–42. 30472399 10.1016/j.adolescence.2018.11.004

[bibr65-01461672221115941] Thomas V. D. Balzer Carr B. Azmitia M. Whittaker S. (2021). Alone and online: Understanding the relationships between social media, solitude, and psychological adjustment. Psychology of Popular Media, 10(2), 201–211.

[bibr66-01461672221115941] Trenberth L. Dewe P . (2002). The importance of leisure as a means of coping with work related stress: An exploratory study. Counselling Psychology Quarterly, 15(1), 59–72.

[bibr67-01461672221115941] Vidal-González P. Fernández-Piqueras R. (2021). Connected solitude: Mobile phone use by Spanish transhumant livestock farmers. Mobile Media & Communication, 9(2), 377–396.

[bibr68-01461672221115941] Wang J. M. Rubin K. H. Laursen B. Booth-LaForce C. Rose-Krasnor L. (2013). Preference-for-solitude and adjustment difficulties in early and late adolescence. Journal of Clinical Child & Adolescent Psychology, 42(6), 834–842. 23682608 10.1080/15374416.2013.794700 PMC3766447

[bibr69-01461672221115941] Weinstein N. Nguyen T. V. T. Hansen H . (in press). With my self: Self-determination theory as a framework for understanding the role of solitude in personal growth. In Ryan R. M. (Ed.), The handbook of self-determination theory.

[bibr70-01461672221115941] Westgate E. C. Wilson T. D. Gilbert D. T. (2017). With a little help for our thoughts: Making it easier to think for pleasure. Emotion, 17(5), 828–839. 28191992 10.1037/emo0000278

[bibr71-01461672221115941] Wichers M. (2014). The dynamic nature of depression: A new micro-level perspective of mental disorder that meets current challenges. Psychological Medicine, 44(7), 1349–1360. 23942140 10.1017/S0033291713001979

[bibr72-01461672221115941] Wilson T. D. Reinhard D. A. Westgate E. C. Gilbert D. T. Ellerbeck N. Hahn C. . . . Shaked A. (2014). Just think: The challenges of the disengaged mind. Science, 345(6192), 75–77. 24994650 10.1126/science.1250830 PMC4330241

[bibr73-01461672221115941] Winnicott D. W. (1958). The capacity to be alone. International Journal of Psycho-Analysis, 39, 416–420. 13610513

[bibr74-01461672221115941] Wood A. M. Linley P. A. Maltby J. Baliousis M. Joseph S. (2008). The authentic personality: A theoretical and empirical conceptualization and the development of the authenticity scale. Journal of Counseling Psychology, 55(3), 385–399.

