# Supplementary material for: Definitions of Solitude in Everyday Life
Source: Pers Soc Psychol Bull. 2022 Sep 3;49(12):1663–78. doi: 10.1177/01461672221115941 (PMC10637102; doi:10.1177/01461672221115941)
Supplement: sj-docx-1-psp-10.1177_01461672221115941 – Supplemental material for Definitions of Solitude in Everyday Life [file sj-docx-1-psp-10.1177_01461672221115941.docx]

Highlights

- This qualitative study identified themes from interviews with adults aged 19-80
- In solitude the self is placed at the center of one's attention
- Solitude has physical or mental distance, and a lack of technology communication
- Solitude benefits from balancing solitude with social time, quiet, and choice
